# Multi-objective optimization of water resources allocation in Han River basin (China) integrating efficiency, equity and sustainability

**DOI:** 10.1038/s41598-021-04734-2

**Published:** 2022-01-17

**Authors:** Lele Deng, Shenglian Guo, Jiabo Yin, Yujie Zeng, Kebing Chen

**Affiliations:** 1 State Key Laboratory of Water Resources and Hydropower Engineering Science, Wuhan University, Wuhan, 430072 China; 2 Bureau of Hydrology, Changjiang Water Resources Commission, Wuhan, 430010 China

**Keywords:** Environmental social sciences, Hydrology

## Abstract

The hydrological cycle, affected by climate change and rapid urbanization in recent decades, has been altered to some extent and further poses great challenges to three key factors of water resources allocation (i.e., efficiency, equity and sustainability). However, previous studies usually focused on one or two aspects without considering their underlying interconnections, which are insufficient for interaction cognition between hydrology and social systems. This study aims at reinforcing water management by considering all factors simultaneously. The efficiency represents the total economic interests of domesticity, industry and agriculture sectors, and the Gini coefficient is introduced to measure the allocation equity. A multi-objective water resources allocation model was developed for efficiency and equity optimization, with sustainability (the river ecological flow) as a constraint. The Non-dominated sorting genetic algorithm II (NSGA-II) was employed to derive the Pareto front of such a water resources allocation system, which enabled decision-makers to make a scientific and practical policy in water resources planning and management. The proposed model was demonstrated in the middle and lower Han River basin, China. The results indicate that the Pareto front can reflect the conflicting relationship of efficiency and equity in water resources allocation, and the best alternative chosen by cost performance method may provide rich information as references in integrated water resources planning and management.

## Introduction

Water resources play key roles in feeding human beings and maintaining sustainable development of a socioeconomic system, which also serve as public resources for the whole society. All members share equal rights to access the resources; whereas, the contradictory relationship between limited resources and increasing demand has gradually come to the fore^1,2^, which gives rise to the water resources allocation issue of efficiency, equity and sustainability^3^. Water resources allocation strategies should not only pursue water consumption efficiency but also consider the equity of water distribution between upstream and downstream, left and right bank as well as water consumption sectors^4^. Meanwhile, facilitating sustainable development is urgent for our social community. Balancing these three factors is the fundamental premise for optimal allocation of water resources, which can effectively maintain healthy social development.

The concepts of efficiency, equity and sustainability are extensively involved in water resources allocation. Efficiency generally refers to achieving highest monetary value for a certain amount of human and material resources as well as capital. From a socio-economic development perspective, highest efficiency is achieved when utilizing limited resources to produce more social wealth and to satisfy human needs^5^. Following sustainable development, the efficiency of water allocation includes economic efficiency, ecological efficiency and social efficiency^6^. However, it’s very difficult to quantify ecological efficiency and social efficiency in water resources systems. Therefore, the safe minimum standard^7^ is adopted to estimate the minimum ecological water demands.

Equity, as another vital index in sustainable socioeconomic development, advocates a fair access to certain living standards and natural resources. This issue has been extensively discussed in social science fields like healthcare and education^8,9^. For instance, Lane et al.^9^ proposed a framework by incorporating an operational definition of equity in healthcare resource allocation, which can facilitate decision-making. There is a growing study focusing on equity in social disciplines; however, less attention has been paid to water-related fields.

From the perspective of water-related research, equity refers to synchronized development across different regions and water consumption sectors^10^. Meanwhile, the economic benefits or the right to utilize water resources should be distributed equally within a basin. It is also important to mitigate the water consumption conflicts between upstream and downstream, as well as that between urban and rural areas. In terms of multi-purpose and versatile consumption, it aims at balancing water consumption in divergent sectors such as domesticity, agriculture and industry^11^. Wang and Palazzo^12^ evaluated the equity performance of sponge city construction in China, which strengthened our understanding of the impacts of stormwater management policies on the social community. Park and Kim^13^ developed a water-energy nexus system in South Korea and examined the regional equity issue within such a system. Despite they employed different approaches to evaluating the equity qualitatively and quantitatively, the optimization solution is not involved. The water-related problems have a large coverage but only few studies have been delved into the equity of water resources allocation. Furthermore, current studies regarding efficiency, equity, or sustainability only focus on one or two aspects and do not consider their interdependence^14–16^, which is insufficient for understanding their complex non-linear relationship. Economic benefit is relatively common in existing water allocation issues, but the equity and sustainability are still poorly understood^17^. For example, Kahil et al.^14^ built an optimization model to maximize farmers' profits in each irrigation district, which might lead to many unavoidable problems. It would cause trouble to allocation equity as well as water utilization sustainability. At present, monetary value is the prominent core of multi-objective optimization in water management^18,19^. However, the equity in water resources allocation was not considered in those studies, and fewer studies have systematically investigated the trade-off between efficiency and equity.

For the third terminology, Harmancioglu et al.^20^ hold that sustainability is a philosophical concept, which is challenging to quantify. In the last few decades, anthropogenic activities have dramatically shifted the natural water resources systems, posing great challenges to ecological environment as well as sustainable development^21^. In recent years, numerous scholars have been dedicated to portraying sustainability on explicit terms, which allowed policymakers to seek relatively sustainable water management practices. Shilling et al.^22^ put forward a framework involving a suite of indicators to measure sustainability relative to targets. Even though it has been evaluated in different facets, the selection of indicator set is still not clear for water resources allocation. Furthermore, it involves complex quantitative operations at different levels, which limits its potential application in this field. Hence, a simpler manifestation of sustainability could be considered.

To fill this knowledge gap, this study attempts to investigate possible solutions for realizing efficient, equitable and sustainable allocation of water resources. A multi-objective optimization model was initially constructed to reconcile the trade-off between efficiency and equity in this domain. The empirical and theoretical investigation implied that substantial inequality would result in deteriorating sustainability^23^. Thus, sustainability was set as a constraint in this model, which is set that the river ecological flow should be satisfied. Economic benefit attends to the total output monetary value of social activities, reflecting the efficiency of the allocation. Gini coefficient, involving comparisons among all variables, is generally employed to assess allocation and income equity^24^. Therefore, it provides an insight to measure the equity of water resources allocation. To achieve a better allocation outcome of synthesis benefits, this study aimed at attaining maximum economic efficiency and allocation equity.

## Materials and data

### Study area

**Figure 1:**
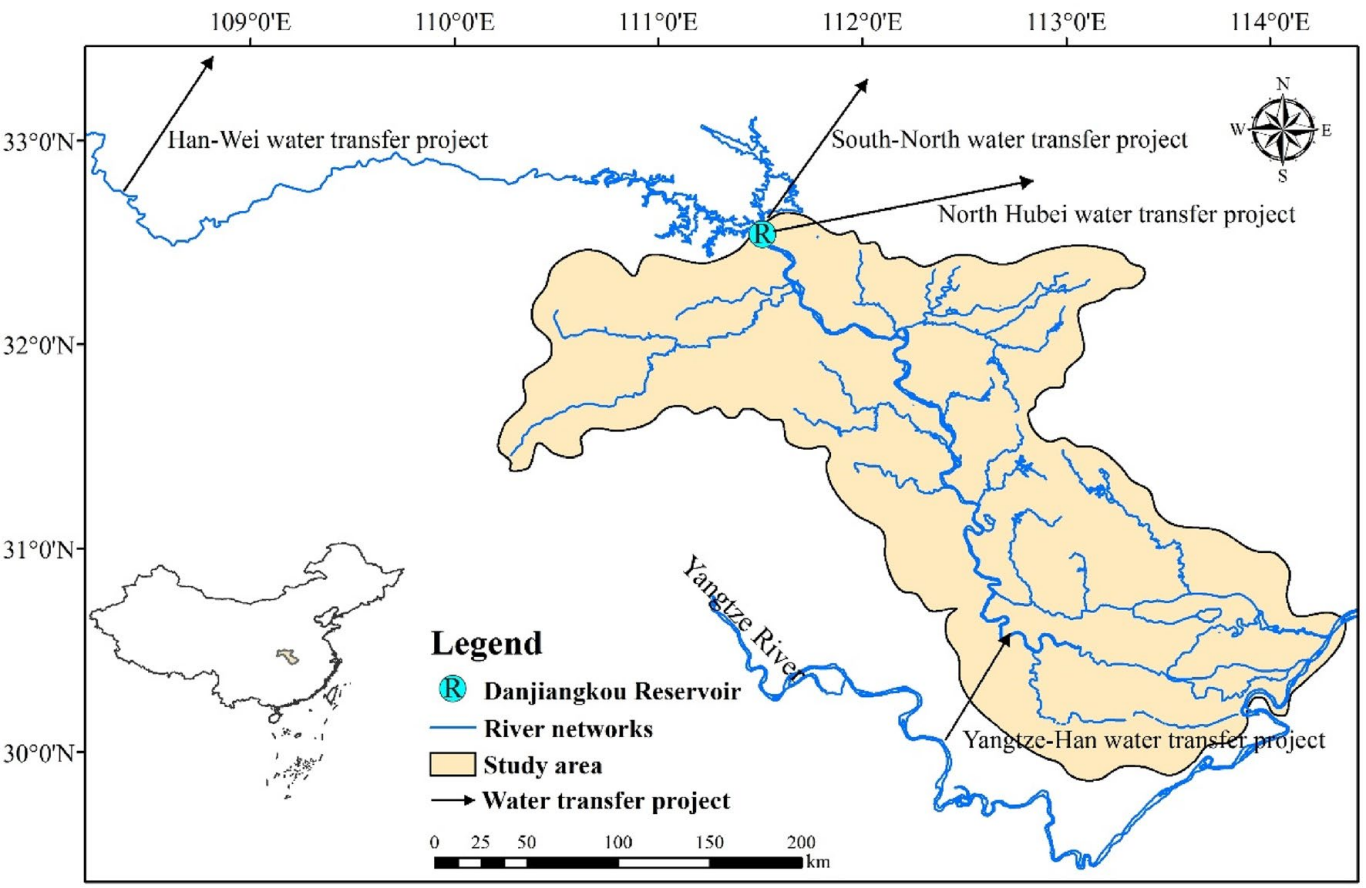
Geographical position and districts in middle and lower Han River basin. (This figure is generated by ArcGIS10.2 software. URL link: http://www.arcgisonline.cn/).

Han River, originating from southern Mountain Qinlin, is the largest tributary of the Yangtze River. It stretches through Shaanxi and Hubei Provinces and feeds the Yangtze River in Wuhan with a total length of 1577 km (Fig. 1). The basin belongs to the East Asian sub-tropic monsoon region and is affected by Eurasia’s continental cold air mass in winter and Western Pacific sub-tropic monsoon in summer, respectively. Thus, the climate has significant seasonality variability. Despite the relative abundance in water resources with perennial annual precipitation, precipitation is mainly concentrated in the wet seasons (May to October), which accounts for 78% of the annual precipitation. The uneven temporal distribution of water resources results in the seasonal water deficit in the basin.

**Figure 2:**
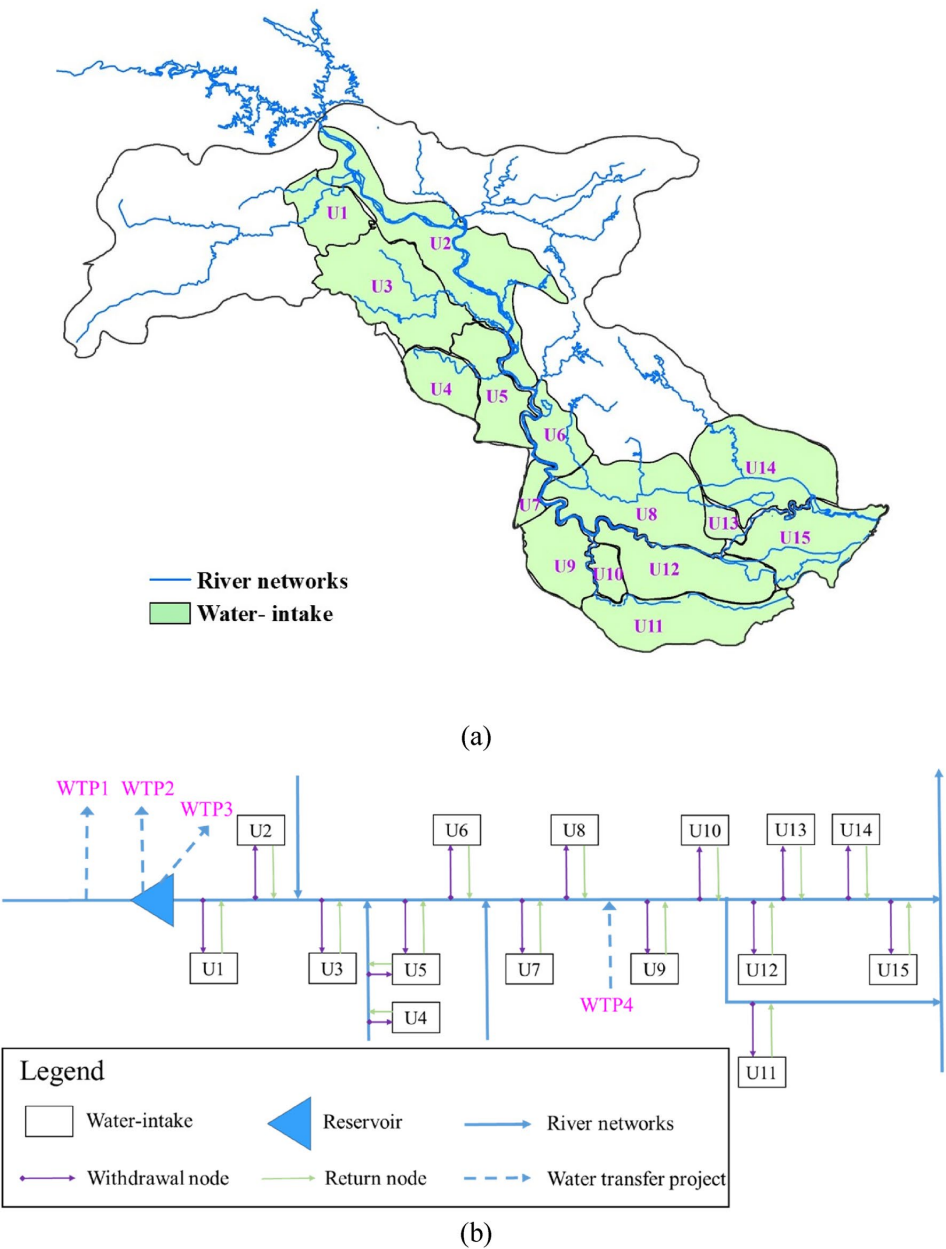
Network of the water supply system of middle and lower Han River basin: (**a**) water-intakes in middle and lower Han River basin [(**a**) is generated by ArcGIS10.2 software. URL link: http://www.arcgisonline.cn/]; and (**b**) schematic diagram of middle and lower Han River basin [(**b**) is generated by Microsoft Powerpoint 2013 software. URL link: https://www.microsoft.com/zh-cn/download/details.aspx?id=55145]. Note: WTP1 refers to Han-Wei Water Transfer Project, WTP2 refers to South-North Water Transfer Project, WTP3 refers to North Hubei Water Transfer Project and WTP4 refers to Yangtze-Han Water Transfer Project; The numbers refer to the water-intake, U1 (Gu-cheng-nan-he), U2 (Shang-you-yin-ti-shui), U3 (Man-he), U4 (Xian-ju-he), U5 (Jing-zhong-you), U6 (Jing-zhong-zuo), U7 (Sha-yang-yin-han), U8 (Tian-men-yin-han), U9 (Xing-long), U10 (Xie-wan), U11 (Dong-jing-he), U12 (Ze-kou), U13 (Chen-hu), U14 (Han-chuan-er-zhan), U15 (Jiang-wei-ti-shui).

The Han River basin can be divided into three sections: (1) upper reach (the upstream of Danjiangkou Reservoir), (2) middle reach (the cross section from the Danjiangkou Reservoir to Huangzhuang), and (3) lower reach (the downstream of Huangzhuang). The middle and lower Han River basin was selected as the case study. As an important agricultural production base in China, this region has complex river–lake networks and is covered by fertile soil, which enjoys fame as “a hometown with fishes and rice”. Several inter-and intra-basin water transfer projects, including the middle route of the South-North water transfer project (SNWTP), North Hubei water transfer project (NHWTP), Han-Wei water transfer project (HWWTP), and Yangtze-Han water transfer project (YHWTP) have influenced the boundary condition in this area. Besides, population growth, rapid urbanization and industrialization have led to many problems concerning equity and sustainability. The study area is important for food production, energy generation and protecting the aquatic environment. To analyze and calculate water resources supply and demand, the network of the middle and lower Han River basin is manifested in Fig. 2a, where the study area is divided into 15 water-intakes. For the convenience of building a mathematic model, a further generalized system is attained (shown in Fig. 2b) according to geographical location and hydraulic connections.

### Water supply and water demand

#### Water supply capacities

This study focuses on several kinds of surface water resources (i.e. mainstream and primary tributary, water conservancy projects, etc.). Water inflow data comprises local water inflow and transferred water from other rivers. The runoff depth is collected from Zhangji, Wanyugou, Yingcheng, Pijiaji, Maliangping, Dagutai, Pijiaji, Wanyugou and Fenxiang hydrological stations. The runoff series with a time step of one month can be calculated by runoff depth and collection area. Likewise, the inflow of reservoirs with various capacities can be obtained. Furthermore, the characteristics of water storage projects such as reservoirs and ponds are listed in Table 1. The local water inflow data period is from May 1956 to April 2016 (a total of 61 years).

**Table 1:** Characteristics of water storage project located in middle and lower Han River basin.

Water-intake	Large-sized reservoirs		Middle-sized reservoirs		Small-sized reservoirs and ponds	
	Area (km^2^)	Storage (million m^3^)	Area (km^2^)	Storage (million m^3^)	Area (km^2^)	Storage (million m^3^)
1			608	4.89	175	5.88
2			389.3	91.8	291	90.59
3	1269	327.44	277	53.71	309	42.81
4			91	25.6	62	11.39
5					20	5.04
6					109.2	53.57
7					307	7.6
8					392.2	46.55
14				44.96	3362	223.84
15					170	35.69

The water-intakes 9–13 are excluded in this table since there are no such facilities in the corresponding area.

#### Water demand

Water consumption coefficient refers to the ratio of water consumption that cannot return to surface water or groundwater aquifer to the gross water supply. Each water-intake includes four water use sectors (i.e. domesticity, industry, agriculture, in-stream ecology), the water consumption coefficients of the first three water usage are 0.608, 0.286 and 0.595, respectively^25^. It is informative to note that the off-stream ecological water demand is contained in the urban domestic water demand.

**Figure 3:**
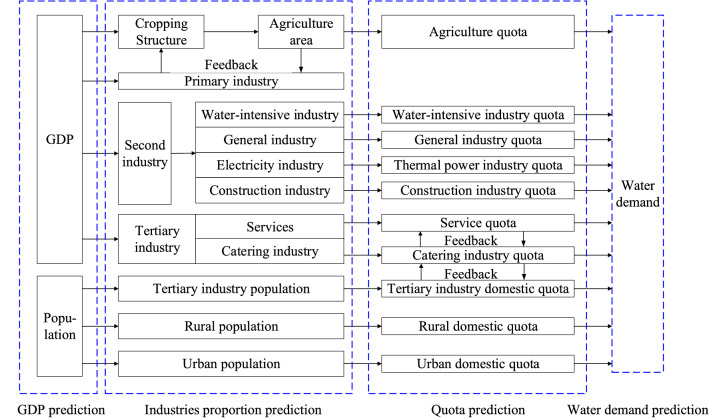
The water demand projection.

The quota method^26^ is applied to estimate the annual water demand in domestic and productive sectors for different periods. The specific illustration of this method employed in this study is shown in Fig. 3. The consumption quota per unit of each water demand category, e.g., water consumption per capita, water consumption per ten thousand Yuan of Gross Domestic Product (GDP) of industry, synthetically net irrigation water requirement per unit area, etc., which are estimated by the economic development and local water management policies. Accordingly, the data of projected urban and rural population, estimated industrial GDP, irrigated area are considered. The in-stream ecological water demand is estimated by the Tennant method^27^.

## Methodology

### Conceptual framework

In this study, we propose a comprehensive water resources management model to define how water can be optimally allocated to achieve the best comprehensive benefit. The proposed model consists of 3 main parts: (1) available water resources and water demand projection; (2) multi-objective optimization of water resources allocation; (3) analysis and evaluation of allocation schemes.

According to hydrological patterns, geographical positions and the requirements of production management departments, 15 water-intakes are segmented in the study. Besides, the water connections, water acquisition and water-break are determined through investigations and official data. Based on the runoff depth, collection area, reservoir operation and inter-basin water transfer projects, the available water resources in each water-intake can be attained. Correspondingly, the water demand of each sector in the planning level year in each water-intake can be predicted.

Furthermore, the optimization objectives are defined in terms of regional sustainable development. The model is supposed to promote high-quality water utilization in objective conditions while tackling the issues of the uneven temporal and spatial water resources distribution, the incoordination between the development of economy and society and the distribution of water resources. Water allocation problems involve complex political, economic, social and environmental elements. Single-targeted water resources distribution does not meet the demand of advanced development of society and economy. Therefore, a water management model needs to address a multi-objective problem.

**Figure 4:**
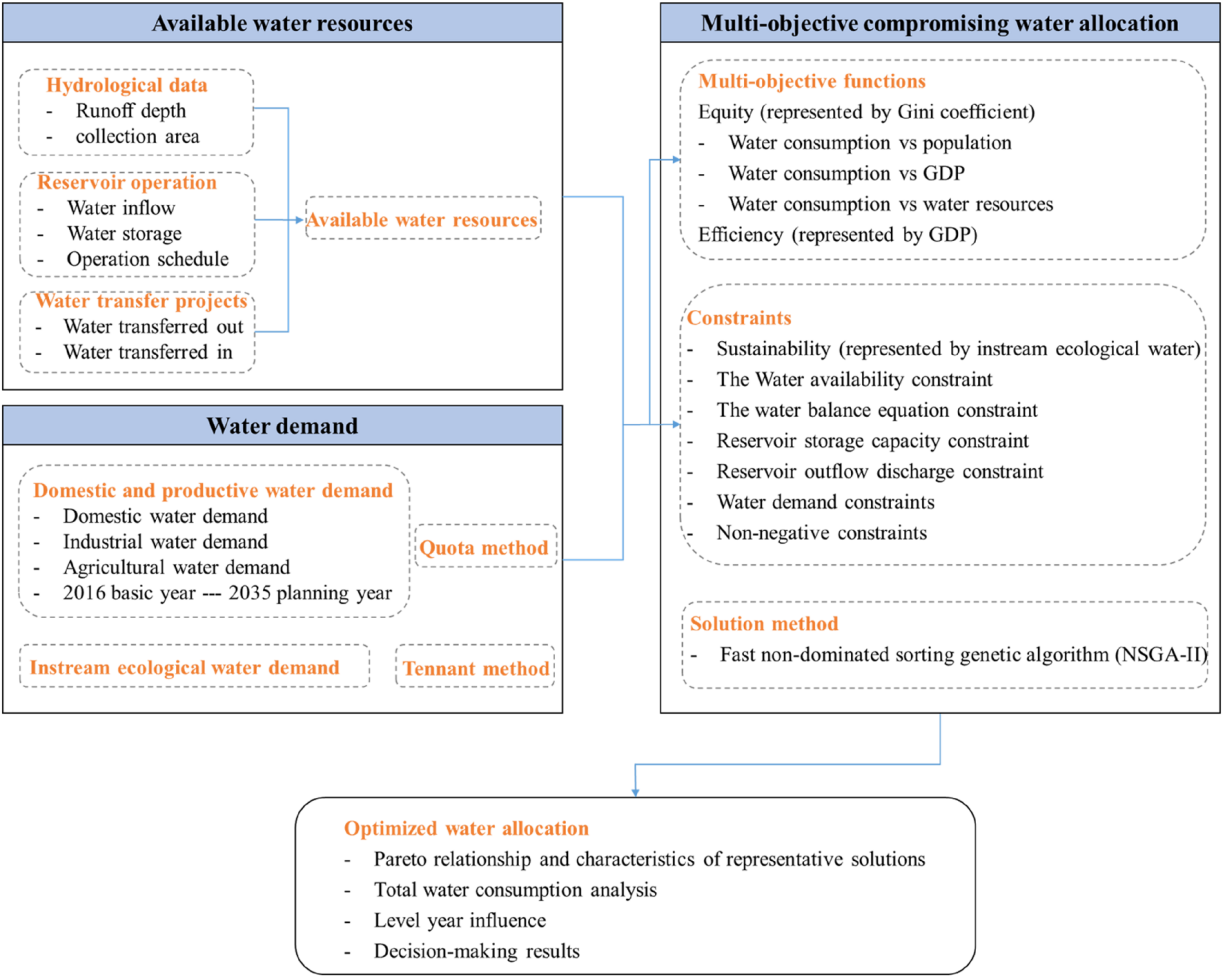
The conceptual framework for optimal water resources allocation.

Taking the above-mentioned development issues into consideration, the general objective of the research is to establish an integrated water allocation scheme for attaining efficiency, equity and ecological sustainability. Under the sustainability constraint, the objectives are maximizing economic interest and water allocation equity. The conceptual framework (Fig. 4) of the proposed methodology, utilized here to identify the optimal quantity of water in the network, demonstrates a practicable mechanism for the appropriate allocation of water resources.

### Optimal water resources allocation model

The ensuing assumptions were declared without conflicts for the water allocation condition in the river basin system before starting the model.

1. The river basin administrative agency takes charge of the water allocation and management, and the water allocated to each water-intake is perceived as tenable and well-grounded.
2. The managers have a profound comprehension as to how the model works, e.g. the objective functions and the constraints, and each water-intake water manager conducts in accordance with the leader.
3. Ecological water is essential for fish, wildlife, water recreation and other related environmental resources. The ecological water demand is necessary to guarantee during the allocation process however economic activities perform^6^. In this study, the minimum ecological water demand is considered for water resources allocation, and the instream flows (ecology sustainability) are guaranteed first and then the remaining water is allocated into different water use sectors and regions, which is a conventional approach in practice^28^. Since the water consumption coefficient of each water use sector is different, and the economic activities are dependent on water availability, the overall water return will be different after economic production activities, and the water consumption of each water use sector in the upstream area will have an impact on the water volume at the outlet section of the area^28^. To meet the sustainability in the process of river water intake, which is set as the constraint in the model, the total water supply to the next area will be affected accordingly. Therefore, from upstream to downstream, the water resources allocation process in the upstream area will affect the water resources allocation in the downstream area.
4. Water-rights trading does not exist in the water-intakes or sectors.

To clearly present the proposed model, the main notations are listed in Table 2.

**Table 2:** The notations in the proposed model .

Category	Notations	Implication
Indices	i	Index of water-intake (i = 1, 2,… 15)
	j	index of water use sector (j = 1, 2, 3, 4)
	t	index of time series (t = 1, 2,… 12)
	max	Superscript of maximum
	min	Superscript of minimum
Parameters	*AW*_i,t_	The available water in *i*th water-intake in *t*th time
	*RWU*_i,t_	The cumulative percentage of water consumption in *i*th water-intake in *t*th
		Time
	*RP*_i,t_	The cumulative percentage of population in *i*th water-intake in *t*th time
	*RG*_i,t_	The cumulative percentage of GDP in *i*th water-intake in *t*th time
	*RW*_i,t_	The cumulative percentage of available water resources in *i*th water-intake in *t*th time
	*PGC*_t_	Gini coefficient between population and water consumption in *t*th time
	*GGC*_t_	Gini coefficient between GDP and water consumption in *t*th time
	*WGC*_t_	Gini coefficient between available water resources and water consumption in *t*th time
	*EPGC*	The average value of *PGC*_t_
	*EGGC*	The average value of *GGC*_t_
	*EWGC*	The average value of *WGC*_t_
Decision variables	*x*	Vector of decision variables $x_{i,j}^{t}$
	*y*	Vector of decision variables $y_{i,j}^{t}$
	$x_{i,j}^{t}$	Water allocated to the *j*th sector in *i*th water-intake in *t*th time
	$y_{i,j}^{t}$	Discharge of the *j*th reservoir in *i*th water-intake in *t*th time

#### Objective functions

*Objective 1* *:* *Maximizing economic efficiency*

Water resources are the vital element for human survival and economic construction. The development of social economy is closely tied to water resources management, which lays the foundation of the former. Humans need to create monetary output as much as possible to seek high-quality development. Consequently, the first objective function is to maximize economic efficiency. For a typical hydrological year with a determined hydrological situation, we neglect the subtle difference between economic efficiency and economic benefit which are coincided generally^29^. The calculation of economic benefit is very easy and has been widely used in complex water resources allocation process. In the real world, a water consumption sector might have feedbacks to other sectors. The specific forward and backward linkages are not clear and quantitative in the state-of-the-art studies. For the simplicity of predicting water demands and allocating water resources, we divide the sectors into domesticity, industry, agriculture and ecology, which has been a general method in water resources planning and management^28,30^. In this study, we selected the gross economic interest to represent the efficiency.

1 $$f_{1} (x) = \max \;\left( {\sum\limits_{t = 1}^{T} {\sum\limits_{i = 1}^{I} {\sum\limits_{j = 1}^{J} {(NER_{i,j} \cdot x_{i,j}^{t} )} } } } \right)$$

where $NER_{i,j}$ refers to the net economic return per unit of water quantity of *j*th sector in *i*th water-intake; $x_{i,j}^{t}$ refers to the water allocated to the *j*th sector in *i*th water-intake in *t*th time. $T$ refers to the total number of months during calculation, *I* refers to the total number of water-intakes, and *J* refers to the total number of water use sectors.

*Objective 2: Maximizing the water allocation equity*

Equity means equal access to water and the benefits of water use. The relative fairness of water use between regions is the basic principle in water resources allocation, and also the core issue of sustainable use of water resources and coordinated development at the regional level. The river authority needs to consider equity in the water allocation process to ensure the balanced development in each water-intake.

Gini coefficient, proposed by Italian economist Gini^31^, has been generally utilized to evaluate the inequality degree in income distribution. In addition to the evaluation of equality in the distribution of wealth in the economic society, it can be applied to various aspects of the assessment of the fairness of distribution in other disciplines, e.g. balance between water and soil^32^.

**Figure 5:**
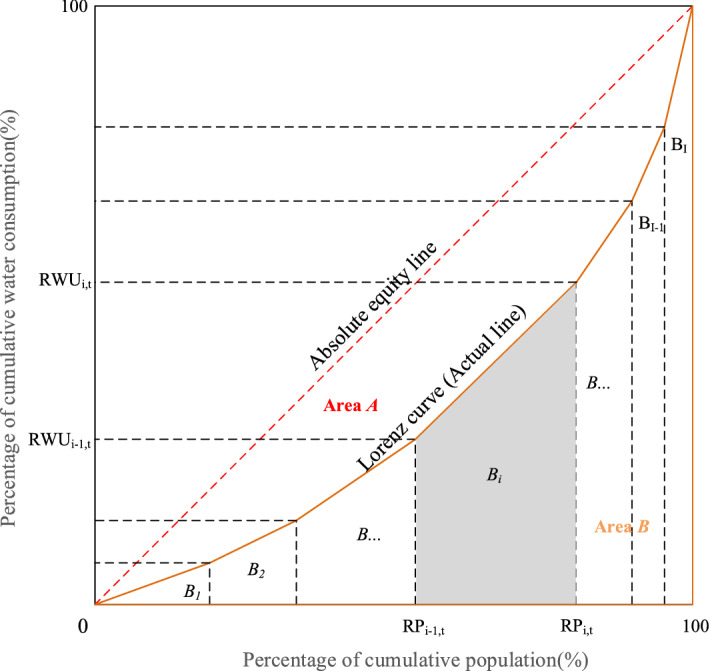
Lorenz curve for water resources allocation.

The Lorenz curve for water resources allocation is presented in Fig. 5, where *X* axis refers to cumulative population share, and *Y* axis denotes cumulative water consumption, *A* represents the area between the absolute equity line and Lorenz curve, and *B* denotes the area below the Lorenz curve. Then the Gini coefficient is equal to *A*/(*A* + *B*)^33^.

In water resources allocation, *Y* axis denotes cumulative water consumption, *X* axis refers to cumulative population share, cumulative gross domestic product share and cumulative available water resources share, respectively. In this study, we use trapezoidal area method to calculate the areas of *A* and *B*. According to the water allocation scheme, we can plot the Lorenz curve for water resources allocation. The value of Gini coefficient is equal to *A*/(*A* + *B*). As *A* + *B* = 1/2, the Gini coefficient is also equal to 1 − 2*B*^3^.

We choose three types of Gini coefficients to evaluate the equality of water resources allocation, i.e., (1) Gini coefficient between population and water consumption (PGC), representing the diversity of per capita water consumption among different regions; (2) Gini coefficient between gross domestic product (GDP) and water consumption (GGC), reflecting the difference between per unit of GDP in different regions; (3) Gini coefficient between available water resources and water consumption, (WGC), denoting water uses’ degree of dependence on other regions. These three kinds of Gini coefficients are calculated as below:

(1) EPGC

2 $$EPGC = \sum\limits_{t = 1}^{T} {\frac{{PGC_{t} }}{T}}$$

3 $$PGC_{t} = 1 - \sum\limits_{i = 1}^{I} {(RWU_{i,t} + RWU_{i - 1,t} ) \cdot (RP_{i,t} + RP_{i - 1,t} )}$$

4 $$EPGC = \sum\limits_{t = 1}^{T} {\frac{{PGC_{t} }}{T}} = 1 - \sum\limits_{t = 1}^{T} {\sum\limits_{i = 1}^{I} {\frac{{(RWU_{i,t} + RWU_{i - 1,t} ) \cdot (RP_{i,t} + RP_{i - 1,t} )}}{T}} }$$

where $EPGC$ denotes the average of $PGC$, $PGC_{t}$ denotes the $PGC$ in *t*th time, $T$ refers to the total number of months during calculation; *I* refers to the total number of water-intakes, $RWU_{i,t} (RP_{i,t} )$ refers to the cumulative percentage of water consumption (population) in *i*th water-intake in *t*th time; $RWU_{i,t}$ and $RP_{i,t}$ are equivalent to zero in the initial stage.

(2) EGGC

5 $$EGGC = \sum\limits_{t = 1}^{T} {\frac{{GGC_{t} }}{T}} = 1 - \sum\limits_{t = 1}^{T} {\sum\limits_{i = 1}^{I} {\frac{{(RWU_{i,t} + RWU_{i - 1,t} ) \cdot (RG_{i,t} + RG_{i - 1,t} )}}{T}} }$$

where $EGGC$ denotes the average of $GGC$, $GGC_{t}$ denotes the $GGC$ in *t*th time, $RG_{i,t}$ refers to the cumulative percentage of GDP in *i*th water-intake in *t*th time, $RG_{i,t}$ is equivalent to zero in the initial stage.

(3) EWGC

6 $$EWGC = \sum\limits_{t = 1}^{T} {\frac{{WGC_{t} }}{T}} = 1 - \sum\limits_{t = 1}^{T} {\sum\limits_{i = 1}^{I} {\frac{{(RWU_{i,t} + RWU_{i - 1,t} ) \cdot (RW_{i,t} + RW_{i - 1,t} )}}{T}} }$$

where $EWGC$ denotes the average of $WGC$, $WGC_{t}$ denotes the $WGC$ in *t*th time, $RW_{i,t}$ refers to the cumulative percentage of available water resources in *i*th water-intake in *t*th time, $RW_{i,t}$ is equivalent to zero in the initial stage.

The second objective function is to minimize the comprehensive Gini coefficient to consider the impacts of various indexes on water consumption.

7 $$f_{2} (x) = \min \;(\omega_{1} \cdot EPGC + \omega_{2} \cdot EGGC + \omega_{3} \cdot EWGC)$$

where $f_{1} (x)$ denotes the comprehensive Gini coefficient, $\omega_{i} (i = 1,\;2,\;3)$ denotes the weighting coefficient and the sum of $\omega_{i} (i = 1,\;2,\;3)$ is equivalent to one. The impacts of population, GDP, and available water resources on water consumption can be viewed as equally important^34^. Hence, $\omega_{i} (i = 1,\;2,\;3)$ are equal to 1/3 in this paper.

#### Constraints

The main constraints are as follows.

(1) Water availability constraints

8 $$\sum {x_{i,j}^{t} } \le AW_{i}^{t}$$

where $x_{i,j}^{t}$ refers to *j*th water use sector in *i*th water-intake in *t*th time, $AW_{i}^{t}$ represents the available water in *i*th water-intake in *t*th time.

(2) Reservoir water balance constraints

For the reservoir, the change of storage capacity is captured by water balance:

9 $$V_{k}^{t + 1} = V_{k}^{t} + (I_{k}^{t} - O_{k}^{t} ) \times \Delta T(t) - L_{k}^{t}$$

where $V_{k}^{t + 1}$ and $V_{k}^{t}$ denote the storage capacity of *k*th reservoir in (*t* + 1)th and *t*th time, respectively; $I_{k}^{t}$ and $O_{k}^{t}$ refer to the reservoir inflow and discharge of *k*th reservoir in *t*th time, respectively. In this study, we also set the discharge of each reservoir as the decision variables, $y_{i,j}^{t}$. $\Delta T(t)$ refers to the time interval; and $L_{k}^{t}$ denotes the water loss of *k*th reservoir in *t*th time.

(3) Reservoir storage capacity constraints

For the reservoir, its operation rule is subject to the physical property and comply with the requirements in practical management, thus the real-time storage capacity is constrained by the maximal and minimal storage capacity:

10 $$V_{k,\min }^{t} \le V_{k}^{t} \le V_{k,\max }^{t}$$

where $V_{k,\min }^{t}$ refers to the lower bound of *k*th reservoir in *t*th time, usually the dead storage capacity; $V_{k,\max }^{t}$ refers to the upper bound of *k*th reservoir in *t*th time, usually the maximum storage capacity below the normal storage water level in the non-flood season and below the flood-control water level in the flood season, respectively.

(4) Reservoir outflow discharge

11 $$y_{i,j}^{t} \le q_{\max i,j}^{t}$$

where $y_{i,j}^{t}$ refers to the discharge of the *j*th reservoir in *i*th water-intake in *t*th time; $q_{\max i,j}^{t}$ refers to the discharge capacity of the *j*th reservoir in *i*th water-intake in *t*th time.

(5) Water demand constraints

For water use sectors in each water-intake, the amount of the water supplied shouldn’t exceed its expectation:

12 $$x_{i,j}^{t} \le Wd_{i,j}^{t}$$

where $Wd_{i,j}^{t}$ denotes the water demand of the *j*th water use sector in *i*th water-intake in *t*th time.

(6) Non-negative constraints

The water allocated to the *j*th water use sector in *i*th water-intake in *t*th time shouldn’t be less than zero:

13 $$\begin{array}{*{20}l} {x_{i,j}^{t} \ge 0} \hfill \\ {y_{i,j}^{t} \ge 0} \hfill \\ \end{array} .$$

#### Global model

In this study, the optimal water resources allocation model can be formulated as shown below:

14 $${\text{Objective}}\quad \quad \left\{ {\begin{array}{*{20}l} {f_{1} (x) = \max \;\left( {\sum\limits_{t = 1}^{T} {\sum\limits_{i = 1}^{I} {\sum\limits_{j = 1}^{J} {(NER_{i,j} \cdot x_{i,j}^{t} )} } } } \right)} \hfill \\ {f_{2} (x) = \min \;(\omega_{1} \cdot EPGC + \omega_{2} \cdot EGGC + \omega_{3} \cdot EWGC)} \hfill \\ \end{array} } \right.$$

15 $${\text{Subject}}\,{\text{to}}\quad \quad \left\{ {\begin{array}{*{20}l} {\sum {x_{i,j}^{t} } \le AW_{i}^{t} } \hfill \\ {V_{k}^{t + 1} = V_{k}^{t} + (I_{k}^{t} - O_{k}^{t} ) \times \Delta T(t) - L_{k}^{t} } \hfill \\ {V_{k,\min }^{t} \le V_{k}^{t} \le V_{k,\max }^{t} } \hfill \\ {y_{i,j}^{t} \le q_{\max i,j}^{t} } \hfill \\ {x_{i,j}^{t} \le Wd_{i,j}^{t} } \hfill \\ {x_{i,j}^{t} \ge 0} \hfill \\ {y_{i,j}^{t} \ge 0} \hfill \\ \end{array} } \right.$$

where $f_{1} (x)$ and $f_{2} (x)$ are the objective functions, $x_{i,j}^{t}$ refer to the design variables and the remaining formulas are the constraints. More detailed information on the programming model can be found in “Objective functions” section and “Constraints” section.

### Optimization algorithm

**Figure 6:**
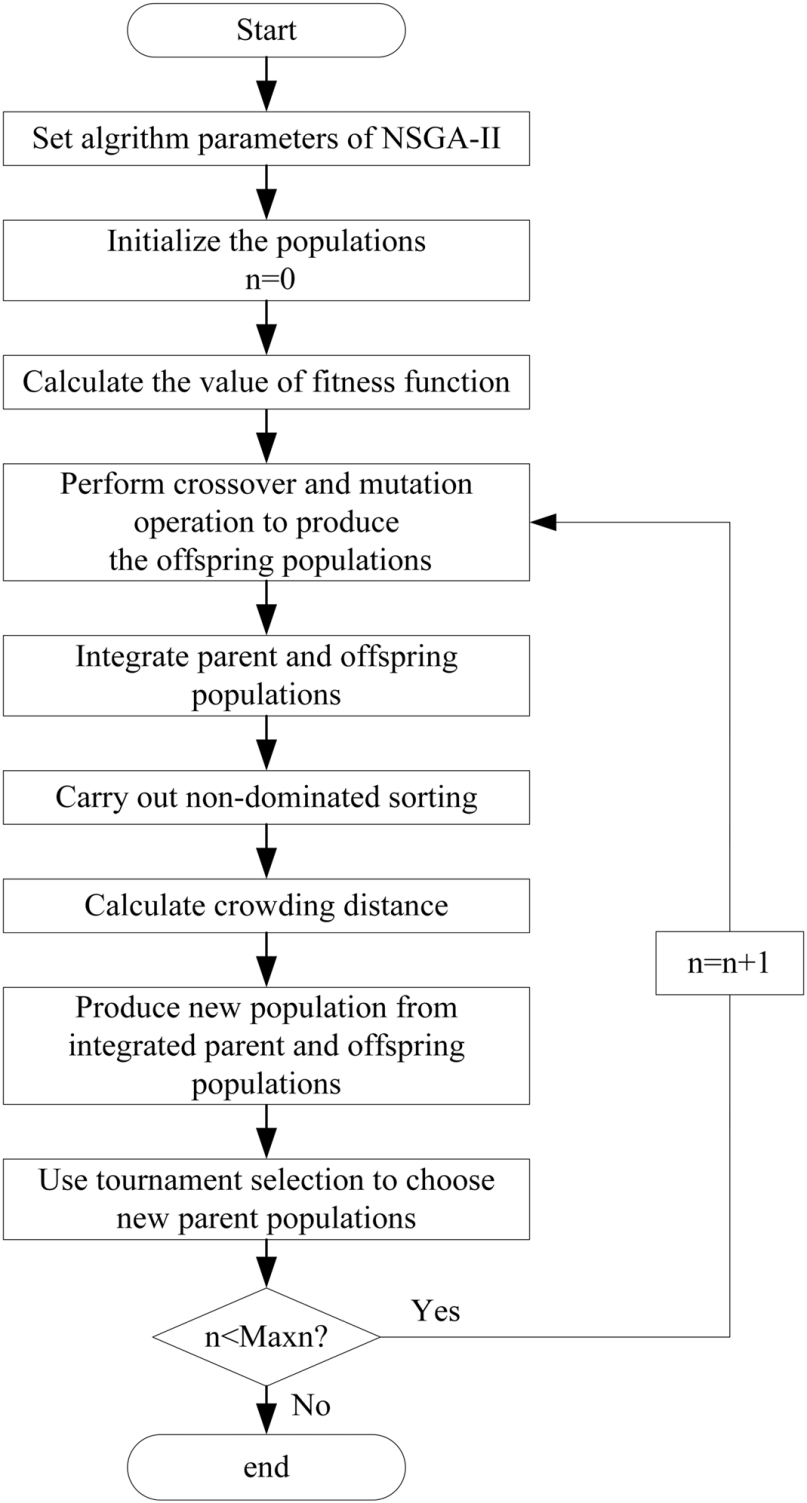
The flowchart of NSGA-II algorithm.

The well-known NSGA-II algorithm (Fast non-dominated sorting genetic algorithm), proposed by Deb et al.^35^, has been extensively applied in multi-objective optimization issues. The employed elitist strategy in this algorithm can preserve the diversity of strategies, so as to enhance the operation speed and improve the robustness^36^. It achieves fast convergence by utilizing the strategies of crowding distance and non-dominated sorting rank^37^. Meanwhile, it reduces the complexity of the traditional non-inferior ranking genetic algorithm and becomes the performance benchmark of other multi-objective optimization algorithms^38^. Due to the above advantages, the evolutionary approach to find the optimal solutions to complex multi-objective optimization problems has been widely used in water resources management^16,34,37–39^. Therefore, NSGA-II is used in this study to trade off efficiency and equity. The flowchart of NSGA-II algorithm is shown in Fig. 6, and the detailed procedure can be found in the reference^35^. The population size, the number of the maximal generation, the crossover probability and the mutation probability were set as 700, 600, 0.9 and 0.1, respectively. It was noticed that the parameters of the NSGA-II could be obtained using an intensive trial-and-error procedure for producing converged results^40^.

### Decision-making method

**Figure 7:**
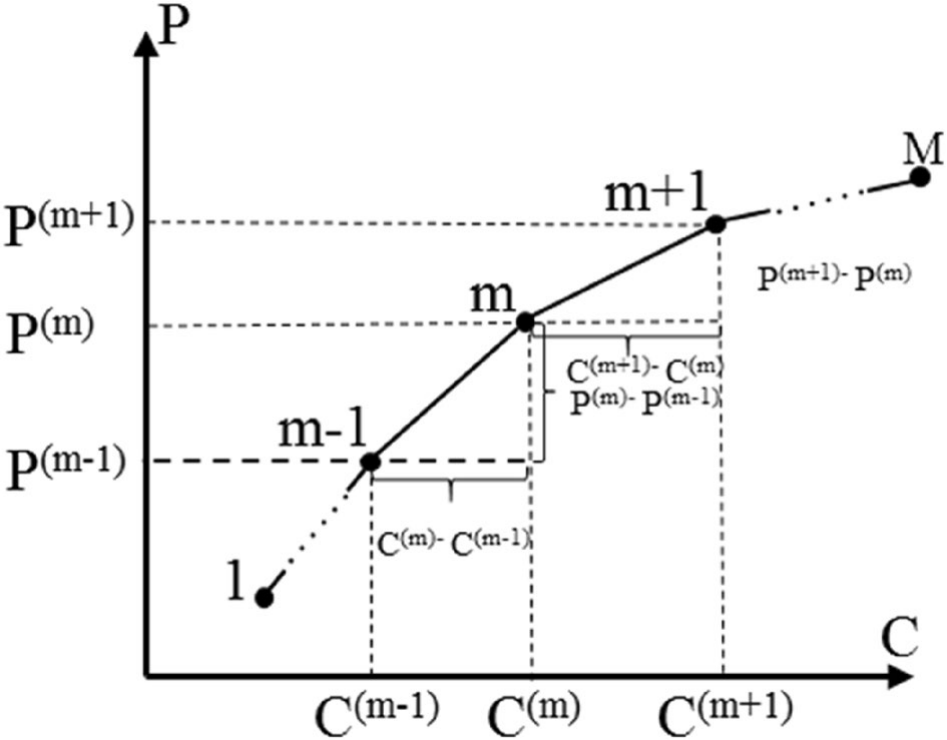
The graphic representation of the average change rate of the objective function values of the Pareto solutions.

Generally, there is a trade-off relationship in the Pareto front. How to select a balance point between optimization objectives is an important issue. To achieve this, we usually need to find a solution that is acceptable to each objective with minimum deviation. The cost performance method proposed by Wang et al.^41^ is utilized to select the solution (Fig. 7).

Let $M$ be the number of Pareto solution sets, which can be sequenced in order. $P^{(m)}$ is the value of the first objective function of the *m*th Pareto solution, $m \in \{ 1,2, \ldots M\}$; $C^{(m)}$ is the value of the second objective function of the *m*th Pareto solution, $m \in \{ 1,2, \ldots M\}$, then the average change rate of the above two objectives is:

16 $$k_{1}^{(m)} = \frac{1}{2}\left( {\frac{{P^{(m + 1)} - P^{(m)} }}{{C^{(m + 1)} - C^{(m)} }} + \frac{{P^{(m)} - P^{(m - 1)} }}{{C^{(m)} - C^{(m - 1)} }}} \right),m \in \{ 2,3, \ldots M - 1\}$$

17 $$k_{2}^{(m)} = \frac{1}{2}\left( {\frac{{C^{(m + 1)} - C^{(m)} }}{{P^{(m + 1)} - P^{(m)} }} + \frac{{C^{(m)} - C^{(m - 1)} }}{{P^{(m)} - P^{(m - 1)} }}} \right),m \in \{ 2,3, \ldots M - 1\}$$

where $k_{1}^{(m)}$ and $k_{2}^{(m)}$ refer to average change rate of the first objective and the second objective, respectively. Especially, when *m is* equal to 1 or M, the average change rate is:

18 $$k_{1}^{(1)} = \frac{{P^{(2)} - P^{(1)} }}{{C^{(2)} - C^{(1)} }}$$

19 $$k_{2}^{(1)} = \frac{{C^{(2)} - C^{(1)} }}{{P^{(2)} - P^{(1)} }}$$

20 $$k_{1}^{(M)} = \frac{{P^{(M)} - P^{(M - 1)} }}{{C^{(M)} - C^{(M - 1)} }}$$

21 $$k_{2}^{(M)} = \frac{{C^{(M)} - C^{(M - 1)} }}{{P^{(M)} - P^{(M - 1)} }}$$

Based on the above results, the sensitivity ratios are calculated by

22 $$\delta_{1}^{(m)} = \frac{{k_{1}^{(m)} }}{{P^{(m)} }},m \in \{ 1,2, \ldots ,M\}$$

23 $$\delta_{2}^{(m)} = \frac{{k_{2}^{(m)} }}{{C^{(m)} }},m \in \{ 1,2, \ldots ,M\}$$

where $\delta_{1}^{(m)}$ and $\delta_{2}^{(m)}$ refer to the sensitivity ratios of the first objective and the second objective, respectively. For the convenience of comparison, the above results need to be dimensionless, the specific expression is

24 $$\varepsilon_{1}^{(m)} = \frac{{\delta_{1}^{(m)} }}{{\sum\nolimits_{m = 1}^{M} {\delta_{1}^{(m)} } }},m \in \{ 1,2, \ldots ,M\}$$

25 $$\varepsilon_{2}^{(m)} = \frac{{\delta_{2}^{(m)} }}{{\sum\nolimits_{m = 1}^{M} {\delta_{2}^{(m)} } }},\;m \in \{ 1,2, \ldots ,M\}$$

where $\varepsilon_{1}^{(m)}$ and $\varepsilon_{2}^{(m)}$ refer to the dimensionless sensitivity ratios of the first objective and the second objective, respectively. The formulas for preference degrees based on sensitivity ratios are as follows:

26 $$\omega_{1}^{(m)} = \frac{{\varepsilon_{1}^{(m)} }}{{\varepsilon_{1}^{(m)} + \varepsilon_{2}^{(m)} }},\;m \in \{ 1,2, \ldots ,M\}$$

27 $$\omega_{2}^{(m)} = \frac{{\varepsilon_{2}^{(m)} }}{{\varepsilon_{1}^{(m)} + \varepsilon_{2}^{(m)} }},\;m \in \{ 1,2, \ldots ,M\}$$

where $\omega_{1}^{(m)}$ and $\omega_{2}^{(m)}$ refer to the preference degrees of *m*th Pareto solution for the first objective and the second objective, respectively.

## Results

### Water demand projection

**Figure 8:**
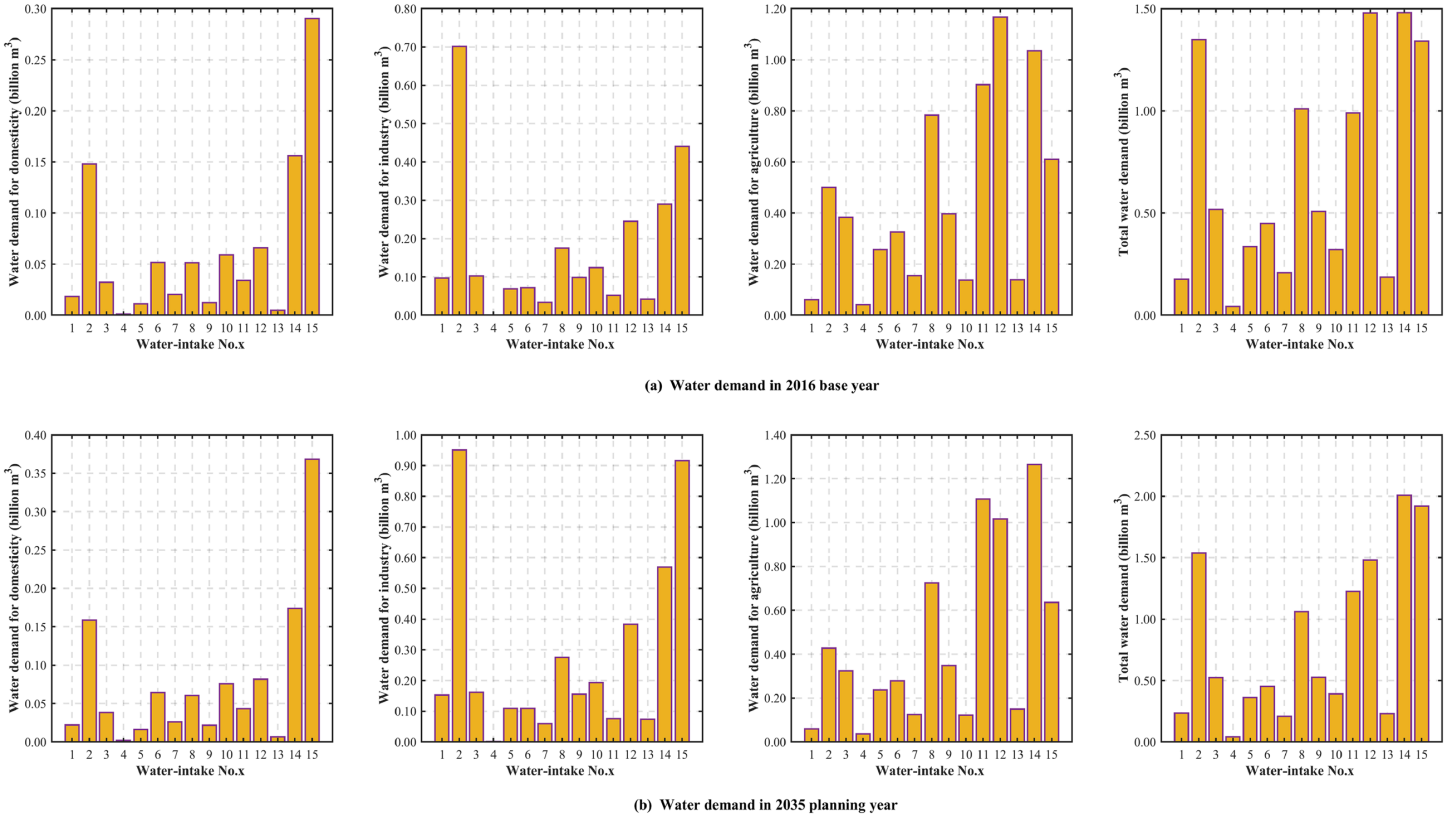
The water demand for each subarea in the 2016 base year and 2035 planning year.

The water resources allocation is usually based on available water for consumption, the lower 75% frequency level of annual water resources was adopted in this study. 1972 was chosen as a typical dry year according to the monthly variability of the water. Based on the quota method in the water demand projection module, the water demand for different water consumption sectors in each water-intake in 2035 planning year is estimated. The water demand in 2016 base year and the projected results for different sectors in 2035 planning year are shown in Fig. 8. The sum of each water demand sector is listed in Table 3.

**Table 3:** Water demand for the study area in 2016 base year and 2035 planning year.

Year	Domesticity (Billion m^3^)	Industry (Billion m^3^)	Agriculture (Billion m^3^)	Off-stream (Billion m^3^)
2016	0.96 (9.21%)	2.54 (24.48%)	6.89 (66.31%)	10.39
2035	1.16 (9.49%)	4.18 (34.30%)	6.85 (56.21%)	12.20

In terms of different water demand sectors, the 2035 planning year witnesses a growth in the water demand for domesticity and industry. The water demand for domesticity increases from 0.96 billion m^3^ in 2016 base year to 1.16 billion m^3^ in 2035 planning year due to the improvement of living conditions and growing population. Due to the saving water program in China, the water use efficiency for the industry will be enhanced in 2035 planning year. Nevertheless, the dramatic rise in industrial economic volume will lead to expansion of water demand for industry (increase by 1.64 billion m^3^). The water demand for agriculture for most water-intakes will fall due to water-saving technologies and crops structure adjustment except water-intakes No. 11 (Dong-jing-he), No. 13 (Chen-hu), No. 14 (Han-chuan-er-zhan) and No. 15 (Jiang-wei-ti-shui), the agricultural water demand will increase due to the expansion of irrigation area. Even though, it shows the decline in the water demand for agriculture in the whole study area (decline by 0.04 billion m^3^).

In 2016 base year and 2035 planning year, the total off-stream water demand for the study area is 10.39 and 12.20 billion m^3^, respectively. Overall, the total water demand shows an increasing trend in 2035 planning year. In 2035 planning year, the water demand in water-intake No. 14 (Han-chuan-er-zhan) is the largest (2.01 billion m^3^) while water-intake No. 4 (Xian-ju-he) requires the lowest water demand (0.04 billion m^3^), from the perspective of different water-intakes. Meanwhile, these two above-mentioned water-intakes rank the same in terms of total water demand in 2016 base year. All of the water-intakes will experience an increase in total water demand, ranging from 0.4 million m^3^ in water-intake No. 7 (Sha-yang-yin-han) to 0.56 billion m^3^ in water-intake No. 15 (Jiang-wei-ti-shui), except water-intake No. 4 (Xian-ju-he). For water-intake No. 4 (Xian-ju-he), the growth in demand for domesticity and industry does not exceed the decline in demand for agriculture, which contributes to the decline in total water demand.

### Pareto relationship and characteristics of representative solutions

The proposed model was utilized to optimize the water allocation schedule monthly for the typical dry year for the middle and lower Han River basin. The runoff time series and water demand series in each water-intake were set as the input of the model, which was solved by NSGA-II algorithm on a server.

**Figure 9:**
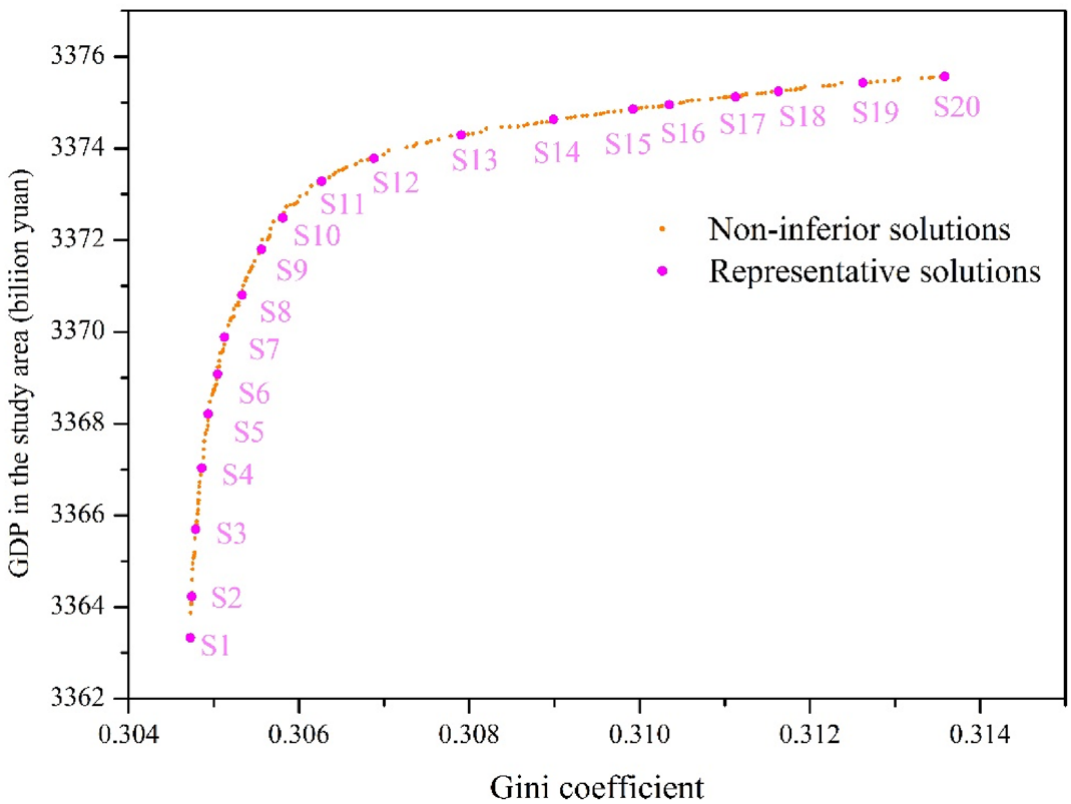
The Pareto frons of the optimal water resources allocation model.

Figure 9 exhibits the optimal Pareto front (Non-inferior solution set) between two objective functions. It indicates that Gini coefficient is positively correlated to efficiency. Commonly, a smaller value of Gini coefficient signifies a better state of water allocation equity, while a greater value of efficiency suggests a more effective utilization of water resources. Hence, the superior efficiency objective matches with the inferior equity objective as well as the value of Gini coefficient increases with the growth of efficiency, translating to a worse condition of equity. The trade-off between equity and efficiency discloses the conflicting nature of equity and efficiency in water resources allocation problems. Policymakers could utilize the solution from optimal Pareto front to coordinate the contradiction between these two objectives and ultimately determine the proper scheme of water allocation following the preference and public requirements. For instance, if the policymakers only allow for achieving the largest gross domestic production, they will incline to choose the scheme which is on the rightmost side of Fig. 9. In another word, the scheme which is on the leftmost side of Fig. 9 could be selected when only equity is taken into consideration.

**Figure 10:**
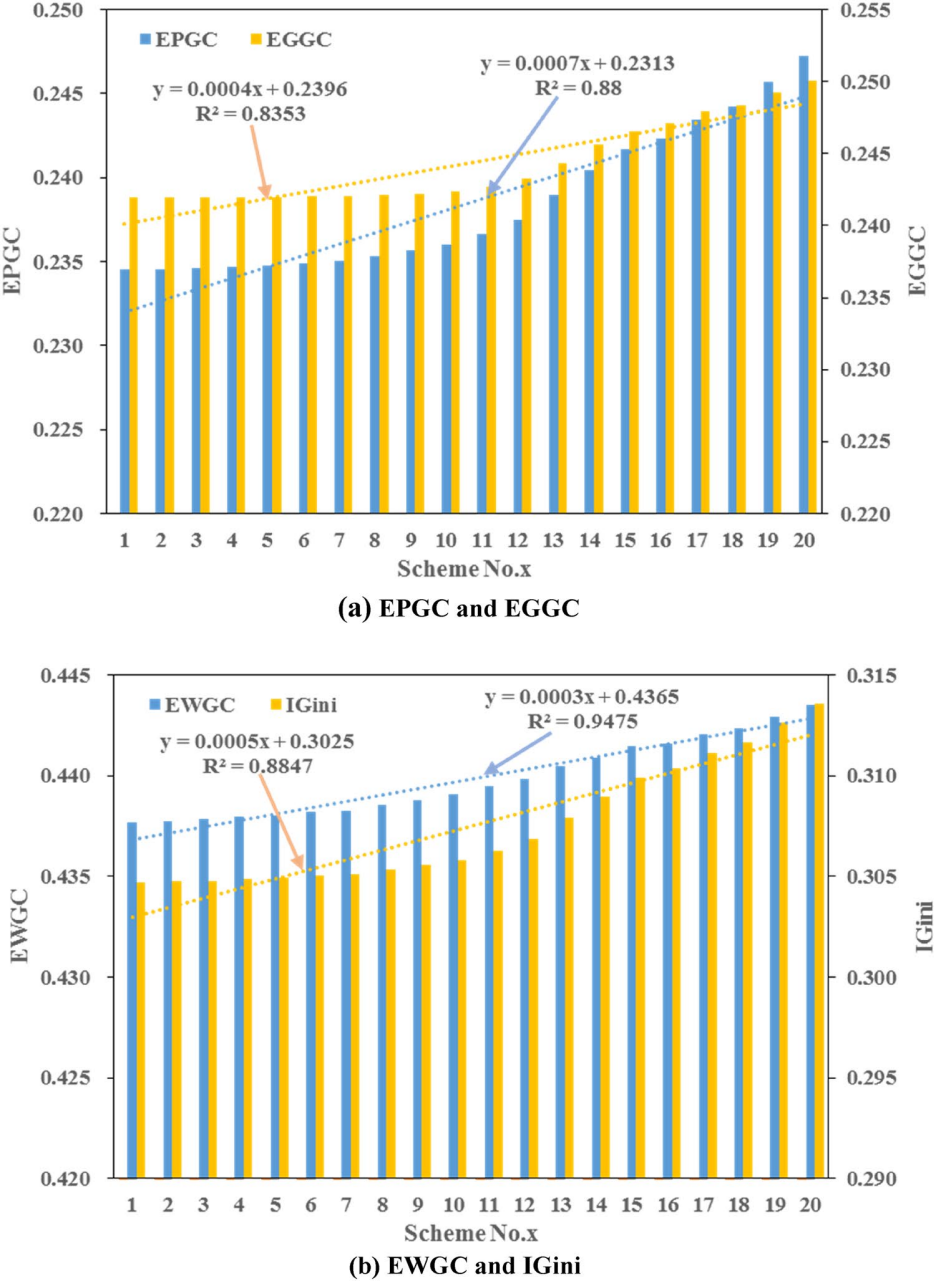
The trend graph of (**a**) EPGC and EGGC, (**b**) EWGC and IGini in middle and lower Han River basin.

20 Pareto set of equity and efficiency objectives with even distribution are taken to analyze the correlation between different Gini coefficients, *i.e.*, EPGC, EGGC, EWGC, and comprehensive Gini coefficient (IGini). The schemes No. 1–20 are sorted by the increasing sequence of efficiency objective, and the trend graphs of EPGC, EGGC are shown in Fig. 10a, and EWGC, IGini are presented in Fig. 10b in the middle and lower Han River basin. Generally, the values of EPGC, EGGC, EWGC, and IGini increase with the upward GDP from scheme No. 1–20 and the correlation between these Gini coefficients and GDP is positive as well. In all schemes, the value of IGini is controlled by the same trend changes of EPGC, EGGC and EWGC since they have the same weighting factors. Meanwhile, the value of EWGC is generally greater than that of EPGC and EGGC, which varied between 0.437 and 0.443 in the Pareto front for all solutions. Hence, the water resources in every water-intake still face great challenges in 2035 planning year.

### Total water consumption analysis

**Figure 11:**
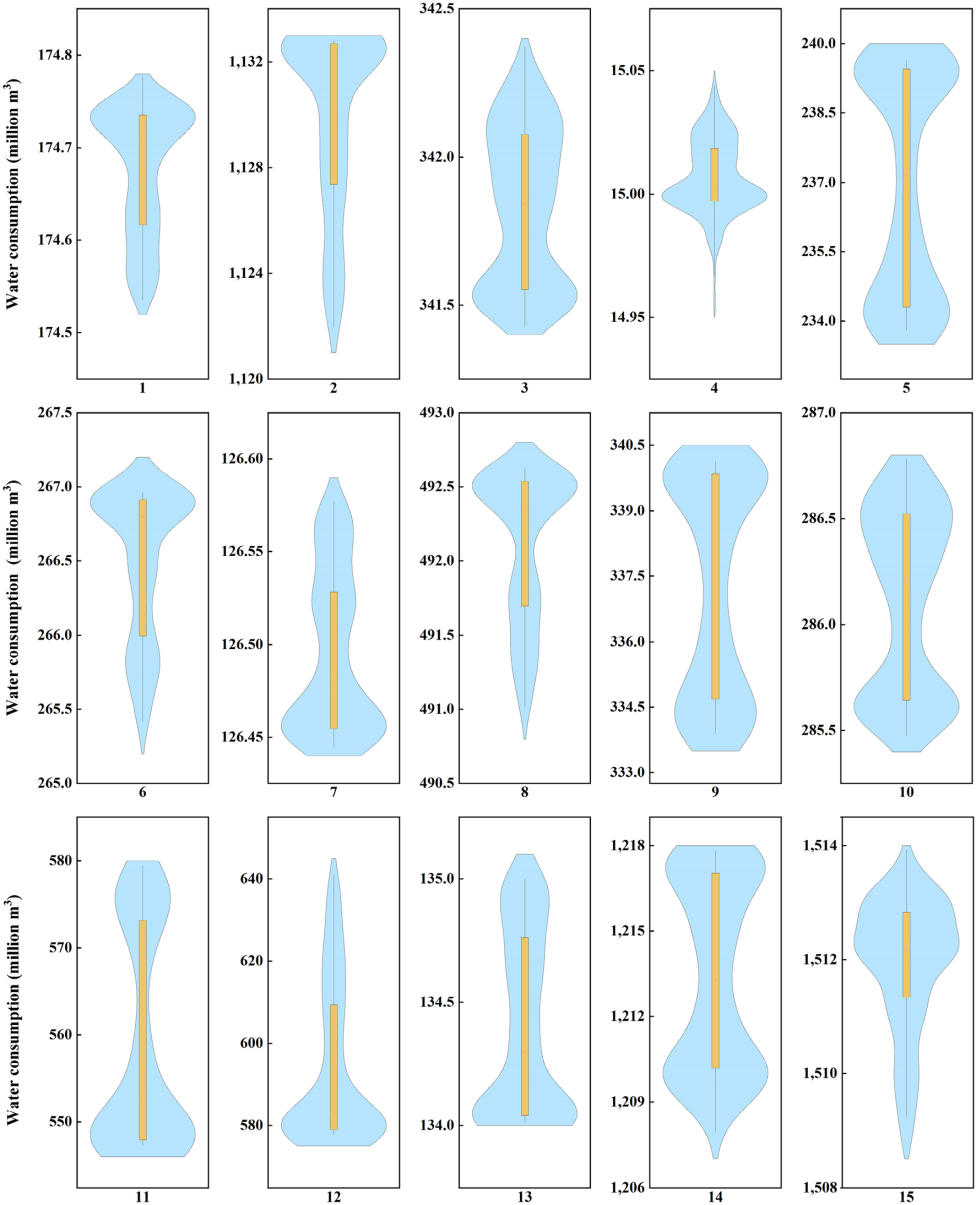
Pareto front values of water consumption.

Table 4 lists the typical values of Pareto front results of water consumption of each water-intake. The distribution results of the Pareto front in different water-intakes in the middle and lower Han River basin are shown in Fig. 11. In the Pareto front, the highest optimization values of water consumption are 174.78, 1132.83, 342.37, 15.04, 239.63, 266.96, 126.58, 492.63, 340.14, 286.78, 579.45, 641.14, 135.00, 1217.82 and 1513.93 million m^3^ for water-intakes No. 1–15, respectively. The lowest optimization values of water consumption are 174.54, 1121.95, 341.43, 14.96, 233.80, 265.41, 126.44, 491.01, 333.89, 285.47, 547.27, 577.74, 134.01, 1207.95 and 1508.87 million m^3^ for water-intakes No. 1–15, respectively. The medium optimization values of water consumption are 174.71, 1131.77, 341.84, 15.00, 237.15, 266.80, 126.47, 492.36, 337.85, 286.22, 552.40, 582.96, 134.29, 1213.32 and 1512.16 million m^3^ for water-intakes No. 1–15, respectively.

**Table 4:** Typical optimization values of water consumption (million m^3^) in the Pareto front.

Water-intake	1	2	3	4	5	6	7	8
Maximum values	174.78	1132.83	342.37	15.04	239.63	266.96	126.58	492.63
Minimum values	174.54	1121.95	341.43	14.96	233.80	265.41	126.44	491.01
Medium values	174.71	1131.77	341.84	15.00	237.15	266.80	126.47	492.36

Water-intake	9	10	11	12	13	14	15	
Maximum values	340.14	286.78	579.45	641.14	135.00	1217.82	1513.93	
Minimum values	333.89	285.47	547.27	577.74	134.01	1207.95	1508.87	
Medium values	337.85	286.22	552.40	582.96	134.29	1213.32	1512.16

It can be seen that the water consumption in water-intake No. 12 ranges from 577.74 million m^3^ to 641.14 million m^3^, with the largest scope of 63.40 million m^3^. Meanwhile, the water consumption in water-intake No. 11 ranges between 542.27 million m^3^ and 579.45 million m^3^. On the contrary, the variation of the water consumption in water-intake No. 4 is the least with the scope of 0.09 million m^3^. The location of water-intake No. 4 is far from the mainstream so it is unable to extract water from the river. It only relies on local water resources to supply for domestic and productive sectors. Moreover, it faces severe drought due to the lower supply capacity of the reservoir, so the total water consumption changes the least. For water-intake No. 11, which lies along the mainstream, exhibits oppositely. According to the systematic hydraulic connection, the amount of the water supply for water-intake No. 11 has a direct impact on the available water resources for water-intakes No. 12–15. Water-intake No. 11 can extract water from the Han River but it returns flow to Yangtze River, which cannot be utilized for the water-intakes lying downstream. For other water-intakes, the flow will return to the mainstream to constitute the available water resources for the next water-intake after the water consumption, which can be computed according to the principle of water balance. In terms of water-intake No. 12, it is subject to the water consumption of upstream water-intake No. 11. The changed situation of water-intake No. 11 could leave a direct impact on the available water for water-intake No. 12. On the other hand, the agricultural water demand is quite large in this area, therefore, the optimization interval is larger.

### Decision-making results

Table 5 lists the minimum and maximum values on the Pareto front for the two objectives. The minimum Gini coefficient value is 0.305 when the total economic interest is 3363.326 billion yuan (Scheme A). The maximum Gini coefficient value is 0.314 when the total economic interest is 3375.567 (Scheme B). It can also reflect that the most superior equity objective matches the most inferior efficiency objective. There is a conflicting relationship between these two objectives. When the study area pursues the highest monetary output efficiency, namely seeking the allocation scheme for attaining the largest economic efficiency, Scheme A is the first choice. When the study area tries to find the best condition of equity, namely seeking the allocation scheme for reaching the minimum Gini coefficient value, Scheme B will stand out.

**Table 5:** Minimum or maximum values (shown in bold font) on the Pareto front for each objective in the water allocation model.

	*f*_1_ (Gini coefficient)	*f*_2_ (billion Yuan)
Scheme A (min. value of *f*_1_)	**0.305**	3363.326
Scheme B (max. value of *f*_2_)	0.314	**3375.567**

However, the water allocation Scheme A or Scheme B only allows for one optimization objective while overlooking the other one, which could not integrate water allocation equity and efficiency. Therefore, we utilize the cost performance method for seeking better decision-making results. Following, 20 representative solutions (i.e., S1 (Scheme A), S2, …, S20 (Scheme B)) from the Pareto front shown in Fig. 9 are evenly selected according to the uniform sampling rule. These solutions can be divided into two categories: category I (i.e., S1 to S10), favor minimizing $f_{1}$; and category II (i.e., S11to S20), favor maximizing $f_{2}$ , where S1 and S20 are two extreme solutions that optimize $f_{1}$ and $f_{2}$, respectively.

**Figure 12:**
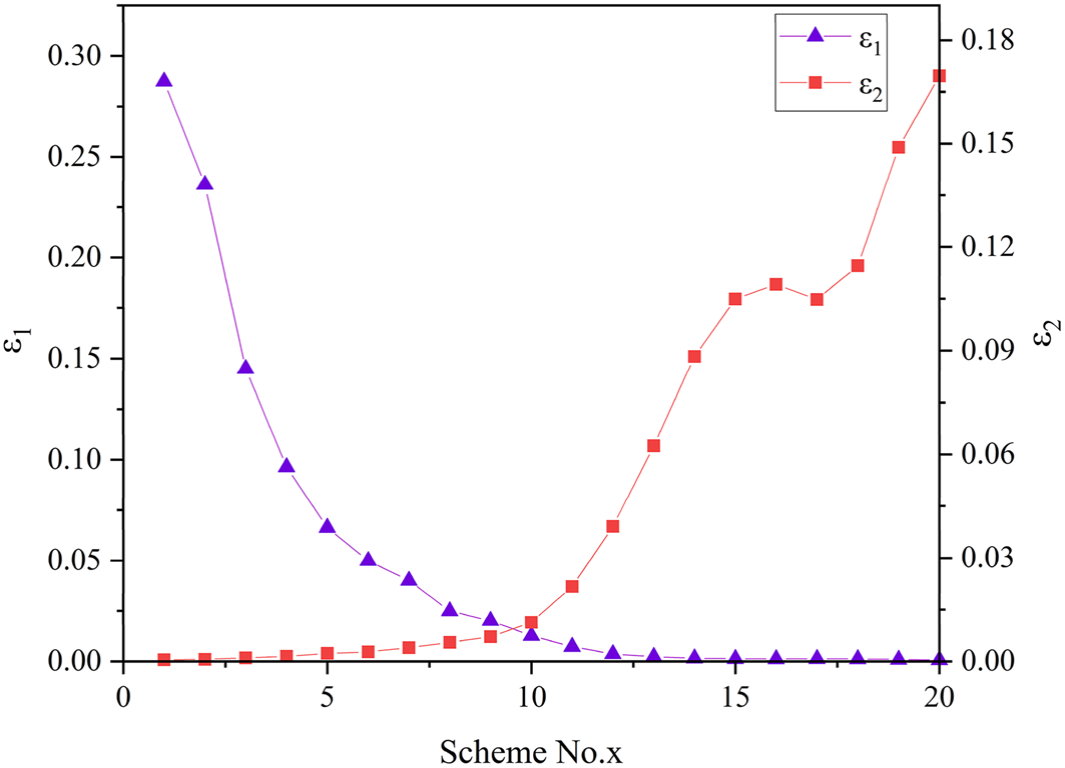
The distribution of dimensionless sensitivity ratio.

The values of the objective $f_{1}$ and $f_{2}$ of the 20 representative solutions are listed in Table 6. Based on the cost performance method, we could calculate the sensitivity ratios of non-dominated solutions corresponding to $f_{1}$ and $f_{2}$, which are listed in the fourth column and fifth column, respectively. After being non-dimensionalized, the distribution of sensitivity ratios is shown in Fig. 12. As listed in Table 6, these parameters are input for the dominance relationship. According to the dominance relationship based on sensitivity ratio, no one solution dominates another solution, hence, all the solutions comprise the new subset of non-dominated solutions. They are set as the input of calculation of preference degree of each Pareto non-inferior solution on different objective functions.

**Table 6:** Parameters of the representative solutions using the cost performance method.

Solution no.	$f_{1}$(Gini coefficient)	$f_{2}$(Billion yuan)	$\delta_{1}$	$\delta_{2} ( \times 10^{8} )$	$\varepsilon_{1} ( \times 10^{2} )$	$\varepsilon_{2} ( \times 10^{2} )$
S1	0.3047	3363.33	166,904.22	0.58	28.74	0.05
S2	0.3047	3364.23	137,160.56	0.75	23.62	0.06
S3	0.3048	3365.70	84,155.81	1.25	14.49	0.10
S4	0.3049	3367.03	55,956.99	1.75	9.64	0.14
S5	0.3049	3368.21	38,472.60	2.83	6.62	0.23
S6	0.3050	3369.08	28,961.14	3.40	4.99	0.28
S7	0.3051	3369.88	23,262.77	4.87	4.01	0.40
S8	0.3053	3370.80	14,446.49	6.73	2.49	0.55
S9	0.3056	3371.80	11,666.03	8.79	2.01	0.71
S10	0.3058	3372.48	7349.73	13.86	1.27	1.13
S11	0.3063	3373.28	4189.33	26.69	0.72	2.17
S12	0.3069	3373.78	2132.87	48.09	0.37	3.91
S13	0.3079	3374.29	1318.58	76.81	0.23	6.25
S14	0.3090	3374.63	900.19	108.49	0.16	8.83
S15	0.3099	3374.86	742.86	128.97	0.13	10.49
S16	0.3103	3374.95	711.52	134.19	0.12	10.92
S17	0.3111	3375.12	740.38	128.79	0.13	10.48
S18	0.3116	3375.24	684.76	140.82	0.12	11.46
S19	0.3126	3375.43	528.00	183.04	0.09	14.89
S20	0.3136	3375.57	452.98	208.55	0.08	16.97

The result of preference degree was listed in Table 7. The quantitative indices could make it more efficient for policymakers for trade-off. For instance, if policymakers favor minimizing $f_{1}$, S1 could be selected for decision-making, the preference degree of which on $f_{1}$ reaches 0.9983 whilst that on $f_{2}$ is only 0.0017. Similarly, if policymakers favor maximizing $f_{2}$, S20 could be selected for decision-making, the preference degree of which on $f_{2}$ is 0.9954 whilst that on $f_{1}$ is only 0.0046. If they weigh two objectives equally, S10 is recommended for policy-makers since the preference degree on $f_{1}$ 0.5288 is closest to that on $f_{2}$ 0.4712 in all non-inferior solutions. The new subset of Pareto non-inferior solutions based on sensitivity ratio could narrow the selection degree as well as provide the quantitative evaluation of the solutions, which is convenient for making decisions.

**Table 7:** The non-inferior solution set based on sensitivity ration with preference degree.

Solution no.	$\omega_{1}$	$\omega_{2}$	Solution No	$\omega_{1}$	$\omega_{2}$
S1	0.9983	0.0017	S11	0.2494	0.7506
S2	0.9974	0.0026	S12	0.0858	0.9142
S3	0.9930	0.0070	S13	0.0351	0.9649
S4	0.9854	0.0146	S14	0.0173	0.9827
S5	0.9664	0.0336	S15	0.0120	0.9880
S6	0.9475	0.0525	S16	0.0111	0.9889
S7	0.9101	0.0899	S17	0.0120	0.9880
S8	0.8197	0.1803	S18	0.0102	0.9898
S9	0.7375	0.2625	S19	0.0061	0.9939
S10	0.5288	0.4712	S20	0.0046	0.9954

## Discussion

This study builds a multi-objective optimization model considering equity, efficiency and sustainability for water allocation, aiming to provide comprehensive water allocation schemes for the middle and lower Han River basin. Despite the superior performance of the multi-objective optimization model, some limitation still remains and several research still needs to be further explored.

### Limitations

Human-water system feedback and interaction are important in characterizing the socioeconomic system. However, these feedback and interaction representations are still lacking in this study due to paucity of data in the study region. We have predicted the water demand in different water-use sectors in 2035 planning year, which holding the assumption that the government policy, economic patterns, or technology is static could contribute to a paradoxical or sub-optimal outcome^42^. This is because it does not focus on the bidirectional feedback between human sub-system and water resources sub-system. One of the challenges of integrating the feedback is to simulate the future political, social and technological scenarios which shape the water demand and water consumption. The rational water resources management requires accounting for human intervention and incorporating human responses to hydrological processes in mathematical models^43^.

In the real word, there are so many sophisticated human agencies involving in the ecosystem. Human (acting individually and collectively) can adapt to the policy and water resources availability and adjust their preference and tactics. In this study, the human agency is represented only by a profit function; which is not sufficient to characterize the complicated system of the human society. Policy-induced or autonomous behavioral changes in human system may affect the hydrologic system, and the feedback further impacts the human system. For instance, more crops of resistance to drought and high efficiency could be planted in case of dry seasons for pursing higher yield and economic output as famers’ perception of water security. Meanwhile, the strategics of land management (i.e., fertilizer), investments in capital stock and other factors all will be influenced^44^. For microeconomic, the adaptive responses are usually driven by nonlinear functions, the outcome of which is related to multi factors in the social economic process. As a result, the water demand and consumption could be altered and consequently the water availability. Another example can be the reshape of the water supply system. The water demand and water supply define the two-way interaction between water system and society system. Human societies are reflexive and respond in unpredictable ways to new information. Greater capital injection can be triggered to upgrade water supply system when water deficit is observed, which in turn matches the population magnitude and urbanization^45^. Therefore, the adaption and feedback should be considered and conceptualized to achieve more holistic understanding of the complicated system.

### Future works

A traditional approach for calculating the economic output incorporated with evolutionary algorithm is adopted for simplicity in this study. However, some ideas or information on the variation of the coefficients and the sectors interplay need to be addressed in the future. We still have a long way to move water resources management as a hydrologic-center discipline towards an integrative and hybrid hydro-economic context. This study adopted piecewise exogenous equations relating water use to economic benefits which has been universally used in contemporary studies^46^. As of yet, this approach overlooked the intrinsic complexity of agents and underestimated the human adaptation to external conditions such as policy interventions or physical alteration. Furthermore, the model inaccuracy or policy design ineffectiveness occurred finally. The resources use efficiency improvements in one sector produce externalities in another, which has been identified in the Coal Question^47^. With respect to the water use, the water consumption of upper stream leaves an impact of water availability of downstream from the perspective of spatial relationship. Similarly, the water consumption of one sector also influences that of other sectors. Meanwhile, complicated interplay exists the economic behaviors among different regions and different sectors. A region can achieve high GDP with more or less water, leaving potential impact on the economic development of other regions. The resources drain or synergetic development remains unknown. Also, the net economic return of food industry will undergo variation if agricultural production experience changes. In a word, the alteration of the external input of the economic model, say water, will exert an influence on the marginal benefit and shadow price, which in turn influences the remaining input variables and feedback to the water subsystem^46^. All of above-mentioned factors need further investigation for better human-water system management.

There is a huge challenge to quantify the uncertainty sourced from different sectors and procedures in the water resources management. For example, the system are involved in muti-factors, including demographic, economic and environmental elements, among others^48^. The deterministic approach show deficiency in revealing the complicated relationship among the numbers of variables. Emerging studies have disclosed that anthropogenic climate change has a direct impact on spatiotemporal variation of precipitation, evapotranspiration and runoff^28^. Meanwhile, population growth, accelerated urbanization, industrial transformation and other factors all pose water resources management to varieties of uncertainties^49^. In this study, we used the traditional approach for forecast of demographic movement, economic development and water demand. However, decision making under deep uncertainty might leave this approach usefulness due to imprecise information as a result of incomplete understanding of the systems and feedback mechanism and other ontological factors. For example, the runoff stochasticity and the fuzziness of agents’ water demands are to be resolved in the future. Some studies have been devoted to integrating the probabilistic information into vulnerability analyses for water resources management^48^, but large gaps still need being bridged to resolve the uncertainty.

## Conclusions

To tackle the issue of efficiency and equity in sustainable water resources management, this study constructed a multi-objective water resources allocation model with maximizing financial benefit efficiency and minimizing Gini coefficient under the sustainability constraint. The model was solved by an intelligent multi-objective algorithm, which identifies the trade-off between the analyzed objectives. The main conclusions were summarized below.

1. In 2035 planning year, the total off-stream water demand of middle and lower Han River basin under 75% annual average water flow frequencies will be 12.20 billion m^3^, which will slightly increase in comparison with 2016 base year. Particularly, there is a huge increase in water demand for domesticity and industry in 2035 planning year.
2. There is a conflicting relationship between efficiency and equity in water resources allocation. The Gini coefficient increases with increasing benefit, leading to a worse condition of equity while a greater monetary value. EPGC, EGGC, EWGC, and IGini increase with the upward GDP and the correlations between these Gini coefficients and GDP are positive.
3. From the Pareto front, the variation of the water consumption in water-intake No. 11 and No. 12 is the greatest while the minimum change occurs in water-intake No. 4. The water resources shortage limits the development of water-intake No. 4 and the water supply of water-intake No. 11 has an impact on the downstream areas, especially for water-intake No. 12.
4. The cost performance method is employed for decision-making, S1 (Scheme A) and S20 (Scheme B) are two extreme solutions that optimize $f_{1}$ and $f_{2}$, respectively. S10 with a minimum gap between two preference degrees is recommended if two objectives weigh equally. This study could be applied to a river basin as a tool to guide the decision-makers to achieve the trade-off between economic development and social equity.
